# DeepVision: Enhanced Drone Detection and Recognition in Visible Imagery through Deep Learning Networks

**DOI:** 10.3390/s23218711

**Published:** 2023-10-25

**Authors:** Hassan J. Al Dawasari, Muhammad Bilal, Muhammad Moinuddin, Kamran Arshad, Khaled Assaleh

**Affiliations:** 1 Electrical and Computer Engineering Department, King Abdulaziz University, Jeddah 21589, Saudi Arabia; hassan.alquid@gmail.com (H.J.A.D.); meftekar@kau.edu.sa (M.B.); 2 Centre of Excellence in Intelligent Engineering Systems, King Abdulaziz University, Jeddah 21589, Saudi Arabia; 3 Department of Electrical and Computer Engineering, College of Engineering and Information Technology, Ajman University, Ajman 346, United Arab Emirates; k.assaleh@ajman.ac.ae; 4 Artificial Intelligence Research Centre, Ajman University, Ajman 346, United Arab Emirates

**Keywords:** artificial intelligence, classification, drones detection, transfer learning, deep learning

## Abstract

Drones are increasingly capturing the world’s attention, transcending mere hobbies to revolutionize areas such as engineering, disaster aid, logistics, and airport protection, among myriad other fascinating applications. However, there is growing concern about the risks that they pose to physical infrastructure, particularly at airports, due to potential misuse. In recent times, numerous incidents involving unauthorized drones at airports disrupting flights have been reported. To solve this issue, this article introduces an innovative deep learning method proposed to effectively distinguish between drones and birds. Evaluating the suggested approach with a carefully assembled image dataset demonstrates exceptional performance, surpassing established detection systems previously proposed in the literature. Since drones can appear extremely small compared to other aerial objects, we developed a robust image-tiling technique with overlaps, which showed improved performance in the presence of very small drones. Moreover, drones are frequently mistaken for birds due to their resemblances in appearance and movement patterns. Among the various models tested, including SqueezeNet, MobileNetV2, ResNet18, and ResNet50, the SqueezeNet model exhibited superior performance for medium area ratios, achieving higher average precision (AP) of 0.770. In addition, SqueezeNet’s superior AP scores, faster detection times, and more stable precision-recall dynamics make it more suitable for real-time, accurate drone detection than the other existing CNN methods. The proposed approach has the ability to not only detect the presence or absence of drones in a particular area but also to accurately identify and differentiate between drones and birds. The dataset utilized in this research was obtained from a real-world dataset made available by a group of universities and research institutions as part of the 2020 Drone vs. Bird Detection Challenge. We have also tested the performance of the proposed model on an unseen dataset, further validating its better performance.

## 1. Introduction

The use of drones for military, commercial, and security purposes is on the rise, as their manufacturing technologies continue to advance, leading to an increase in their numbers [1,2]. The efficacy of using various types of drones in airport security, facility protection, and integration with surveillance systems has garnered significant interest in the past few years [3]. Conversely, drones can pose a substantial risk in these security domains, underscoring the need to devise an effective method for detecting different drone varieties in such applications [4]. These technologies have the potential to safeguard airports and military installations against drone incursions or breaches, thereby enhancing their overall security [5]. Hence, it is imperative to discuss public safety and the potential risks posed by unmanned aerial vehicles (UAVs) by focusing on their detection, recognition, and identification. Detection involves observing a target, which may appear dubious and jeopardize the security of the surrounding environment. Recognition entails ascertaining the category to which the target belongs, while identification involves identifying the specific type of target category.

The main goal of this article is to detect and identify a specific drone variant by highlighting similarities in their physical attributes and behaviors to those of birds. Additionally, the research aims to distinguish this type of drone from birds. Various sensors have been utilized in literature for identical and detection purposes, including radar [6], LIDAR [7], and RF-based sensors [8]. Moreover, acoustic [9] and thermal [10] sensors have also been employed for drone detection and recognition. Nonetheless, the majority of such sensors are expensive and consume substantial amounts of energy [11]. Furthermore, the integration of drones with these sensors is restricted by their weight and size, and thermal sensors typically have lower resolution. Despite the absence of challenges related to incorporating detectors and unmanned aerial vehicles, employing optical imaging systems offers greater detail than infrared systems. However, challenges such as obscured regions, congested backgrounds, and inconsistent illumination in drone-captured images do exist. Consequently, the effectiveness of addressing these concerns depends on the particular approach used for detecting and distinguishing the aerial device. Identifying and discerning drones from birds is often challenging due to various factors, such as: (1) the similarities in physical appearance and behaviors between drones and birds, causing confusion, especially at long distances; (2) obstacles in detection due to environmental conditions, such as weather, crowds, and lighting variations; and (3) the difficulties in identifying small-sized drones at longer distances, resulting in challenges in recognition.

Deep learning networks have emerged as the most effective models for visual processing, particularly in tasks such as object detection and tracking [12]. Over the past decade, there has been significant interest in using these networks for object detection, as they offer greater accuracy and computational power than alternative approaches [13]. Deep neural networks, particularly convolutional neural networks (CNNs), have been widely regarded as among the most effective approaches for object recognition [14]. CNNs excel at feature extraction, making them a popular and extensively researched choice for object recognition [15]. Due to their superior ability to extract features, CNNs are preferred over traditional object recognition methods [16]. Therefore, a research study was carried out to assess the ability of CNNs to detect drones in visible images. Four different CNN models were utilized, namely ResNet-18, ResNet-50, MobileNet-V2, and SqueezeNet. ResNet-18 and ResNet-50 are particularly popular due to their ability to handle large datasets and complex features. MobileNet-V2 is a lighter-weight architecture designed for mobile devices, while SqueezeNet is a compressed architecture that provides good accuracy with fewer parameters. The purpose of using these specific models was to evaluate their individual strengths and weaknesses in identifying drones in images that are invisible to the human eye.

Detecting and recognizing drones are crucial for a variety of applications, including public safety. Visible imagery is an ideal sensor for this purpose due to its high resolution, low cost, and compatibility with different drones. However, this approach presents challenges, such as crowded backgrounds and confusion between drones and birds due to their similar sizes. To address these issues, a suitable method must be employed. Deep networks based on transfer learning are known for their ability to provide high accuracy and speed in analyzing input images [17]. Transfer learning involves a pre-trained model as a starting point and then fine-tuning it for a specific task. This approach enables the model to leverage knowledge gained from large datasets and use it for a new task, resulting in improved accuracy and faster training times. Transfer learning is particularly useful when working with limited training data, as it allows the model to learn from a broader dataset without requiring as much data for training. Therefore, transfer learning-based deep networks are an effective and efficient solution for image analysis tasks, including object detection, recognition, and classification. The objectives of this study were: (1) to develop an efficient and effective deep network for UAV detection; (2) to evaluate the performance of four popular CNN architectures, including ResNet-18, ResNet-50, MobileNet-V2, and SqueezeNet, for recognizing UAVs utilizing visible imagery; and (3) to achieve accurate and real-time identification of UAVs, with various practical applications, such as security, surveillance, and search and rescue operations. Our main contributions are as follows:

(1) The proposed method is tested on a real-world dataset made available by a group of universities and research institutions as part of the 2020 Drone vs. Bird Detection Challenge;
(2) The proposed method is able to detect and identify a specific drone variant by highlighting similarities in their physical attributes and behaviors with those of birds;
(3) Given that drones can appear to be incredibly small in relation to other flying objects, we presented a robust image-tiling technique using overlaps that performed better in the presence of very tiny drones;
(4) An innovative deep learning method is proposed to effectively distinguish between drones and birds, the main features of which are as follows:
  (a) The proposed method is based on the SqueezeNet model, which has less computational complexity than the best-performing ResNet18 model for the task of drone detection;
  (b) The proposed SqueezeNet-based method has shown better AP scores, faster detection times, and more stable precision-recall dynamics;
  (c) The proposed method is more suitable for real-time implementations because it has less computational architecture and a faster detection time;
(5) The proposed method’s performance is further validated by testing its detection performance on an unseen dataset.

## 2. Materials and Methods

### 2.1. Dataset Description

The training dataset utilized for the Drone vs. Bird Detection Challenge comprises 77 distinct videos, each consisting of an average of 1384 frames, with an average of 1.12 annotated drones per frame. Among these videos, 11 were collected from the previous iteration of the challenge through MPEG4-coded static cameras by the Fraunhofer IOSB research institute and the SafeShore project. In 2020, the ALADDIN project contributed 45 additional videos in three different resolutions, namely 1920 × 1080 @25 fps, 720 × 576 @50 fps, and 1280 × 720 @30 fps. These videos were recorded at two different locations, each with distinct geographic characteristics, and they feature seven types of drones, including three with fixed wings and four rotary ones. Most of the videos are short, lasting around one minute, with the exception of two videos that are five minutes long. The remaining 21 training videos were supplied by Fraunhofer IOSB and were recorded at various locations throughout Germany, featuring five types of rotary drones. In total, the dataset consists of videos showcasing eight different types of drones.

In general, the training dataset demonstrates a high degree of variability in terms of difficulty. This variability results from the videos encompassing a diverse set of backgrounds, including sky or vegetation, and various weather conditions, such as sunny and cloudy weather. Additionally, the dataset also incorporates direct sun glare and variations in camera characteristics. Another aspect that adds to the complexity of the dataset is the varying distance of the drones from the camera, which can differ significantly within and across videos. As a result, there is a substantial variation in the sizes of drones. Drone sizes can range from as small as 15 pixels to more than 1,000,000 pixels. The majority of annotated drones in the dataset are relatively small, with sizes ranging from fewer than 162 pixels to 322 pixels. The small size of the drones in many of the videos makes the detection task even more challenging. The dataset includes both static and moving camera recordings. Each video is accompanied by a separate annotation file containing the frame number and the bounding box (expressed as [topx topy width height]) for the frames in which drones enter the scene. Some of the videos also feature birds, which are not individually annotated and can be a significant source of interference.

The evaluation dataset comprises an additional 14 videos that lack accompanying annotations. These videos exhibit characteristics akin to those in the training dataset, with some shared locations. However, not all the locations are identical. Within the evaluation dataset, three video sequences feature a moving camera. To prevent any single video from disproportionately influencing the evaluation results, longer sequences have been truncated to one minute. Access to the dataset requires signing a data utilization agreement (DUA), and the annotations can be found at https://github.com/wosdetc/challenge, accessed on 14 April 2021.

### 2.2. Split Dataset and Software

The videos were separated into two distinct groups for training and validation by the authors through a manual process. The primary objective of grouping was to maintain independence between the two groups to the greatest extent possible. The division was performed on the basis that videos recorded at identical locations must be included in the same group. The validation group was then chosen by selecting 16 videos from the split. The majority of the videos used for this study were captured at high frame rates, which led to a significant number of identical images. To address this issue, a subsampling technique was performed by selecting every 10th frame, resulting in a reduction of the dataset to only 10% of the original data. This smaller dataset was then used for training and validating the model.

Matlab 2022b software and deep learning modules were employed for the analysis. The experiments were conducted on a PC with an Intel Core i7-7500U processor running at 2.70–2.90 GHz and 12 GB of RAM. For the classification task, ResNet18, ResNet50, MobileNetV2, and SqueezeNet modules from the TensorFlow library, version 2.6.2, were utilized, all of which can be downloaded for free.

### 2.3. Image Preprocessing

This section details the dataset used for training and the methods used for image tiling and data augmentation. The primary dataset utilized to train and test the approach was the “2020 Drone vs. Bird Challenge”. Under the leadership of Alexis, the team removed one annotated sequence from the available 77, resulting in a total of 106,485 frames. Although the dataset includes both birds and drones, the annotations are limited to drones only. To further enhance the dataset, 26,500 synthetic images were added through the method outlined in the “Cut, Paste and Learn” paper [18]. To create these images, roughly 100 close-up images of birds and drones were sourced from Google Images, and object masks were manually generated. The objects were then blended onto outdoor backgrounds through various methods. In the synthetic dataset, annotations for bounding boxes were limited to drones only, unlike the Drone vs. Bird dataset, in which both drones and birds were annotated.

One of the key challenges in drone detection is the possibility of drones being exceptionally small, often appearing as merely 10–20 pixels compared to a full high-resolution image. Downsizing images before processing them through deep convolutional neural networks can be ineffective in detecting such small drones, as they may become too insignificant to differentiate from other aerial objects. As a solution, image tiling has proven to be a highly effective strategy [19], albeit at the cost of longer processing times. The image tiling approach involves dividing the input image into multiple patches or tiles. To enhance robustness and redundancy, overlaps may be included between tiles, which can be especially useful in scenarios in which drones appear on the borders of a tile.

During the training procedure, random cropping was utilized as a means of emulating image tiling. In 80% of cases, the cropping location was chosen in a way that ensured that at least one drone was included. In all other cases, the crop location was chosen randomly. This strategy helped to provide more instances of positive drone examples while also providing background examples. To improve the training process, the team led by Alexis utilized various data-augmentation techniques. These techniques included affine transformations, flips, blurring, noise, brightness adjustments, and several other augmentations sourced from the Albumentations package [20].

### 2.4. Pre-Trained Deep Networks

**Figure 1:**
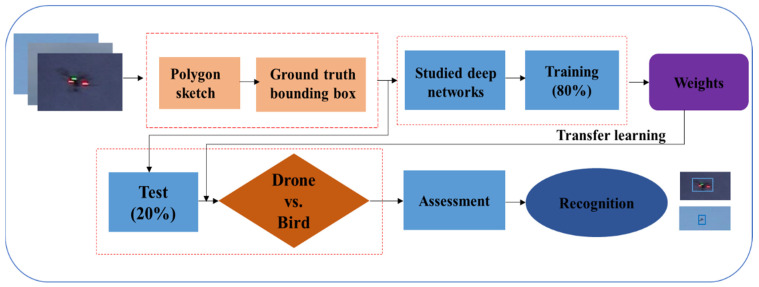
The proposed detection and recognition diagram using different deep networks.

This article proposes a deep learning-based method for detecting and recognizing drones despite the challenges posed by crowded backgrounds, resemblances to birds, small sizes at longer distances, and lighting inconsistencies. The proposed approach involves four main steps, as shown in Figure 1. The first step involves preparing the input data for the proposed architecture. In the second step, a network training phase is implemented to detect and recognize two types of drones, as well as birds. The third step involves testing the trained model on a large variety of drone and bird datasets. Finally, the model’s performance is evaluated, and the detection and recognition process is conducted on the input test data.

#### 2.4.1. Resnet18

ResNet-18 is a deep neural network architecture that was introduced by Microsoft Research in 2015 as a part of their research on deep learning. It is a variant of the ResNet (Residual Network) family of neural networks, which are known for their ability to train very deep neural networks utilizing residual connections [21]. ResNet-18 consists of 18 layers, including a convolutional layer, followed by four blocks of convolutional layers, and it ends with a fully connected layer. Each of these blocks contains two or three convolutional layers and a shortcut connection, which allows the network to learn residual functions. The use of residual connections in ResNet-18 allows for easier training of very deep neural networks by enabling the network to learn an identity mapping if needed, effectively bypassing a set of layers if it deems it necessary. In this way, the gradient can flow more easily through the network, and the network can learn to model more complex features. ResNet-18 has been widely used in various computer vision tasks [22], such as image classification, object detection, and segmentation. It has achieved state-of-the-art performance on many benchmark datasets, including ImageNet, CIFAR-10, and CIFAR-100.

#### 2.4.2. Resnet50

ResNet is an advanced deep learning architecture consisting of fundamental residual blocks that serve as a connection across layers. This connection permits the training of hundreds or thousands of layers while simultaneously enhancing performance. ResNet was the winner of the 2015 Imagenet classification and is a dependable solution for many computer vision problems [23]. The number accompanying ResNet denotes the number of deep layers, with ResNet50 having 50 processing layers. The network’s size is 96 MB, with 25.6 million parameters. The ResNet50 architecture accepts input in 256 dimensions and features skip connections, which address the vanishing gradient descent problem. Each ResNet begins with convolution and max-pooling operations, followed by stacked convolutions. This layered convolution approach provides advantages in resolving the vanishing gradient problem [24].

#### 2.4.3. MobileNetV2

The network based on depthwise separable convolutions was initially developed for mobile and embedded vision applications, as presented by Howard et al. [25]. Compared to other common models, deep convolutional neural networks, such as MobileNet, are more compact and faster. Each input is filtered by a single filter using depth-wise independent convolutions before being combined into a collection of output features through 1 × 1 convolutions. Depth-wise separable layers are similar to traditional convolutional layers, but they work much faster and with less modification. In depth-separable convolution, the standard filters and assemblies are separated into two layers: one for filtering and one for mixing. This process reduces the model’s size and computational power requirements. Batch normalization and ReLU nonlinearity are applied to all layers in this network, except the last fully connected layer, which feeds into a softmax layer for classification without nonlinearity. MobileNet’s convolutions include 28 layers, excluding the depthwise and pointwise convolutions. The network has a size of 5.2 MB and contains 1.3 million parameters.

#### 2.4.4. SqueezeNet

SqueezeNet is a convolution network that executes better performance than AlexNet with 50× fewer parameters [26,27]. SqueezeNet consists of 15 layers with five different layers as two convolution layers, three max pooling layers, eight fire layers, one global average pooling layer, and one output layer softmax. SqueezeNet is a deep neural network architecture designed for efficient computation and small memory footprints. It was introduced by researchers at the University of California, Berkeley, in 2016. SqueezeNet uses a combination of techniques, such as filter factorization, 1 × 1 convolutions, and aggressive downsampling, to reduce the number of parameters while maintaining high accuracy. Compared to ResNet-18, SqueezeNet has a much smaller memory footprint and requires fewer computations, making it more suitable for deployment on devices with limited computing resources, such as drones. However, the trade-off is that SqueezeNet may not be as accurate as larger and more complex models, such as ResNet-18.

### 2.5. Evaluation Metrics

To assess the effectiveness of the proposed method, a range of evaluation metrics were used, including Intersection over Union (*IoU*), precision, and recall [28,29]. This evaluation strategy allowed for a more comprehensive understanding of the model’s performance to be gained. The *IoU* metric measures the extent of overlap between the predicted bounding box and the ground truth bounding box. In the study, the optimal threshold for classifying the input data was identified by plotting the true positive rate against the false positive rate for various thresholds and choosing the threshold that maximizes the area under the curve. The chosen threshold was 0.5, which was used to classify the input data. If the *IoU* value exceeds 0.5, the classification is considered a true positive (TP); otherwise, it is classified a false positive (*FP*). The confusion matrix was constructed using the numbers of *TP*, *FP*, *TN* (true negative), and *FN* (false negative) values.

**Figure 2:**
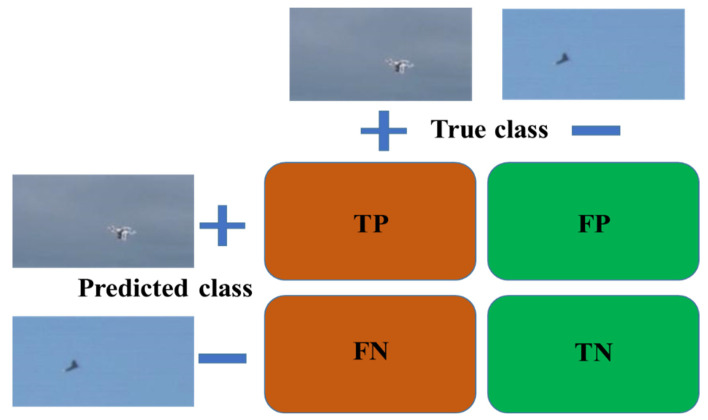
Confusion matrix of the proposed framework.

The confusion matrix (Figure 2) is a fundamental tool for evaluating model accuracy and is typically of size *n* × *n*, where *n* is the number of classes [30]. In this study, the columns of the confusion matrix represent the true class of intended objects, including two types of drones and birds. Meanwhile, the rows indicate the predicted classes generated by the proposed deep learning model. The positive class pertains to drones, while the negative class relates to birds. Since this study involves two classes, the confusion matrix is generalized to a size of 2 × 2. Precision and recall are computed using the following equations:

(1) $$IoU=\frac{TP}{FP+TP+FN}\times 100$$

(2) $$Pr=\frac{\sum TP}{\sum TP+\sum FP}\times 100\;$$

(3) $$Re=\frac{\sum TP}{\sum TP+\sum FN}\times 100$$

## 3. Results and Discussion

### 3.1. Ablation Study for Selection of SqueezeNet Hyperparameters

To determine the best hyperparameters empirically, an ablation study was performed using SqueezeNet as the baseline model. The findings of this study are reported in Table 1. The results are reported around the MiniBatch size, L2 regularization, and Momentum values of 2, 0.0005, and 0.9, respectively, yielding the best results. 

### 3.2. Assessment of SqueezeNet and ResNet18 Performance

**Figure 3:**
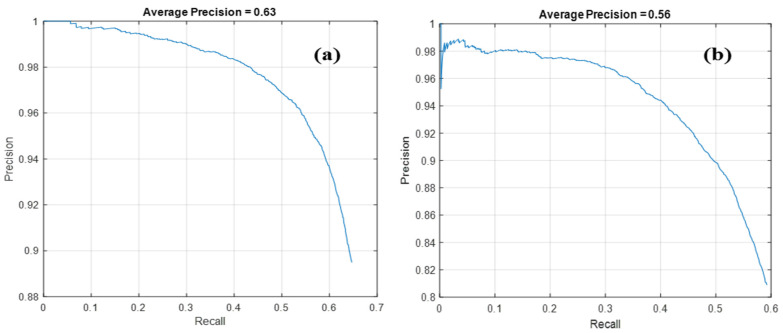
The precision–recall relationship of proposed deep networks: (**a**) SqueezeNet; and (**b**) ResNet18.

Table 2 presents a comparison of the performance of the deployed deep learning models, including SqueezeNet and ResNet18, across different overlap thresholds, area ratios, and training and detection times. A thorough investigation was conducted on the average precision (*AP*) for the range of overlap thresholds between 0.5 and 0.9. Additionally, the *AP* values were calculated for three different area ratios, namely small, large, and medium. The results unequivocally point to the SqueezeNet-based network, fortified by the use of data-augmentation techniques, as the most efficient in drone detection during the evaluation process, a finding also corroborated by Schmidhuber [31]. Specifically, SqueezeNet achieved an AP of 0.327 for overlap thresholds ranging from 0.5 to 0.9. This outcome is significant when contrasted with the ResNet18 model, which registered a comparatively lower *AP* value of 0.237 within the same range of overlap thresholds. Further granularity is provided when we look at area ratios. For medium-sized area ratios, the SqueezeNet model surpassed ResNet18 by securing an *AP* of 0.770 compared to ResNet18′s *AP* of 0.739. Although both models performed robustly for medium area ratios, SqueezeNet’s edge in this category could make it the preferred choice for specific drone-detection scenarios requiring that particular area ratio. Moreover, when it comes to detection time—an essential factor in real-time, critical applications—SqueezeNet was superior again, clocking in at a mere 0.061 s. This time is significantly shorter than the ResNet18 model’s time of 0.105 s, underscoring the computational efficiency of the former. Figure 3 deepens our understanding by illustrating the precision–recall relationship for these two architectures. As the recall rate increased, SqueezeNet maintained high precision levels, fluctuating between 100% and 88%. Meanwhile, ResNet18′s precision saw a larger degree of variance, ranging from 98% to 80%. These findings reinforce the importance of top-level network features in the effectiveness of drone detection. In summary, SqueezeNet’s superior *AP* scores, faster detection times, and more stable precision-recall dynamics indicate that it may be more suitable for real-time, accurate drone detection than ResNet18.

### 3.3. Performance Analysis of ResNet50 and MobileNetV2

**Figure 4:**
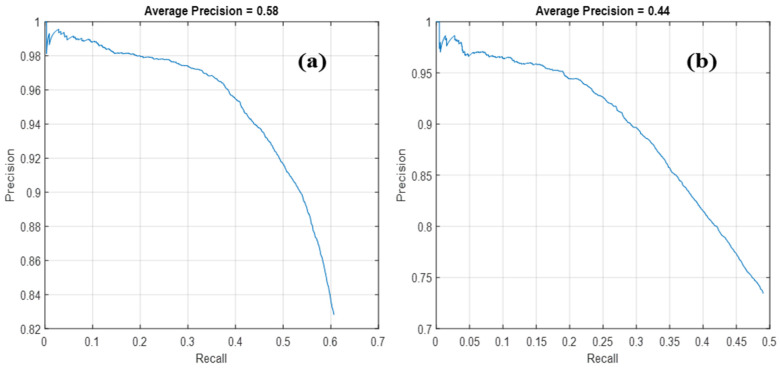
The precision–recall correlation of the proposed deep networks: (**a**) ResNet50; and (**b**) MobileNetV2.

Table 3 offers a comprehensive look at the comparative performance of two deep learning models, ResNet50 and MobileNetV2, focusing on various evaluation metrics, including different overlap thresholds, area ratios, and both training and detection times. At a glance, the results highlight the superiority of ResNet50 over MobileNetV2 when it comes to efficiently detecting drones. Diving deeper into the data, for the specified overlap thresholds spanning from 0.5 to 0.9, ResNet50 garnered a notable *AP* value of 0.254. In contrast, MobileNetV2 lagged behind, managing an *AP* score of only 0.166 within the same overlap threshold range. Such a significant disparity in scores underscores the refined efficiency of ResNet50 in these particular evaluation conditions. This pattern of dominance by ResNet50 continues when we factor in the area ratios. Particularly with medium area ratios, ResNet50 outpaced MobileNetV2. The former achieved an *AP* score of 0.610, while the latter settled for a score of 0.485. Such distinctions in performance can be pivotal, especially in applications in which accurate detection in medium-sized areas is of paramount importance. However, it is not all smooth sailing for ResNet50. In terms of sheer speed, MobileNetV2 showcased a detection time of a brisk 0.111 s, edging out ResNet50, which clocked in at 0.129 s. This outcome hints at the architectural efficiency of MobileNetV2, making it potentially more suitable for scenarios in which rapid detection is more crucial than utmost precision. Figure 4 further amplifies our understanding by offering a visual representation of the precision–recall dynamics for both models. As recall values rose, ResNet50 demonstrated precision ranging between 100% and 74%. MobileNetV2, while exhibiting a slightly broader fluctuation, maintained precision levels between 100% and 80%. Crucially, these findings serve as a testament to the significance of hyper-parameter tuning in the training phase. For instance, judiciously setting overlap thresholds and area ratios can critically enhance a model’s efficacy in drone detection. The sentiment aligns perfectly with the insights from the research by Redmon and Farhadi [32]. Their work spotlighted the instrumental role of selecting optimal hyperparameters, especially overlap thresholds and area ratios, in fine-tuning the performance of object detection models. This role remains true irrespective of the target detection object, but it is particularly vital in specialized tasks such as drone detection, in which precision can be mission-critical.

### 3.4. Proposed Deep Networks’ Learning Curves

**Figure 5:**
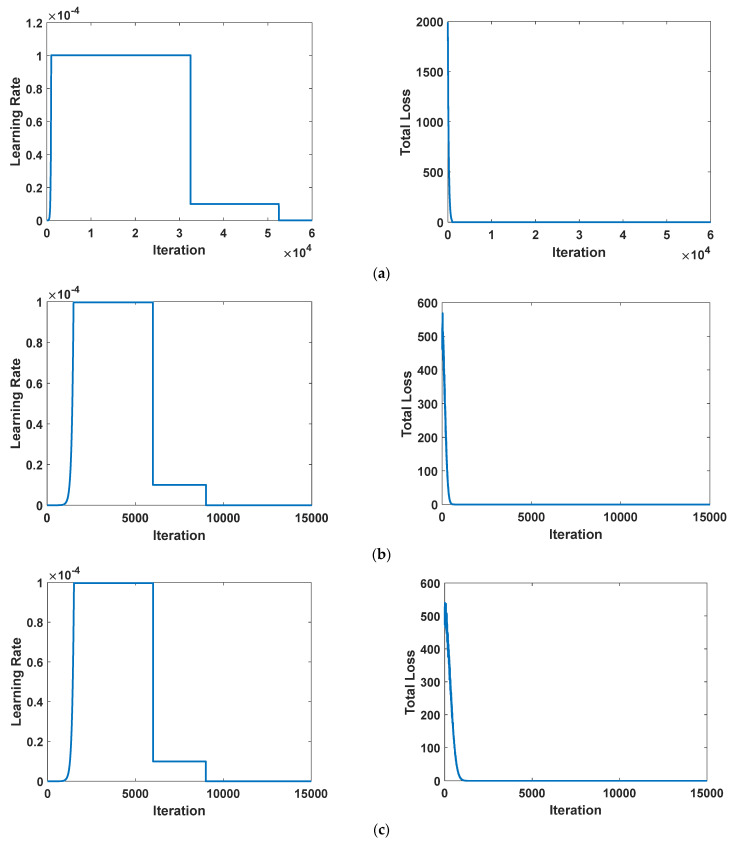

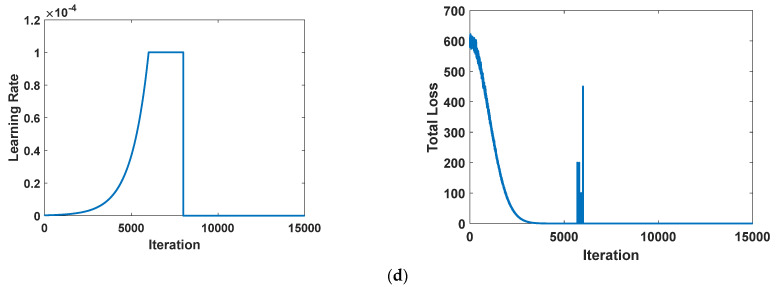
The associations among learning rate, total loss, and iteration for the suggested networks: (**a**) SqueezeNet; (**b**) ResNet18; (**c**) ResNet50; and (**d**) MobileNetV2.

Several investigations were conducted to enhance the learning curve of the super recognition model. These investigations included: (1) examining the properties of the selected premium features obtained by training a specific ML model [33]; (2) selecting optimal factors, including overlap thresholds and area ratios; (3) applying data augmentation techniques; and (4) training the deep network with early stopping to prevent overfitting. These efforts have culminated in the development of a high-performance deep network architecture. The enhanced SqueezeNet network surpassed other suggested networks, displaying reliable true positive and true negative values without any false negatives or false positives, making it effective in drone identification. As illustrated in Figure 5, it demonstrates the behavior of advanced models with iterations during training on the dataset. As the number of iterations increases, the learning rate gradually increases during training until a point is reached at which the learning curves indicate a high-quality model. Simultaneously, the loss of the models gradually decreases as the number of iterations increases. As depicted in Figure 5a–d, the performance of SqueezeNet, ResNet18, ResNet50, and MobileNetV2 improved after 5, 9000, 9000, and 6500 iterations, respectively. This study’s results match those of Elsayed et al. [34], who employed early stopping during the training of different deep networks to mitigate overfitting, leading to an anticipated enhancement of the expected performance of the models.

### 3.5. Performance Comparison

**Figure 6:**
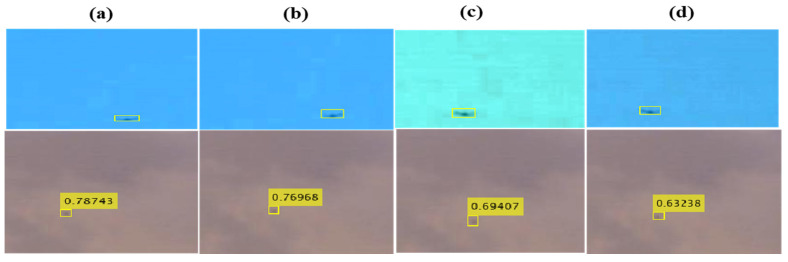
Visual samples displaying qualitative detection outcomes alongside their corresponding ground truth annotations (yellow) with different proposed models: (**a**) SqueezeNet; (**b**) ResNet18; (**c**) ResNet50; and (**d**) MobileNetV2.

This section presents an assessment of the method’s effectiveness, in which experiments were conducted to demonstrate the influence of various hyper-parameters, including tile size and data-selection and augmentation approaches, which can yield significant effects. The evaluation employed the AP, which serves as the standard measure for object detection. The results displayed in Table 4 correspond to a test dataset consisting of images obtained through a varied methodology. Based on the AP scores presented in the table, SqueezeNet achieved the highest AP score of 0.770, indicating a strong ability to accurately classify the images in the test set. MobileNetV2 and ResNet18 achieved lower AP scores of 0.485 and 0.739, respectively, suggesting a relatively weaker performance compared to SqueezeNet. Interestingly, ResNet50 demonstrated an AP score of 0.610, which falls between the scores of MobileNetV2 and ResNet18. This outcome indicates that ResNet50 performed moderately well in classifying the images. Moreover, it is worth noting that the AP score reported by Coluccia et al. [35] in their study was significantly higher, at 52.2%. This finding implies that their approach, which is not explicitly mentioned in the provided information, outperformed all the methods examined in the current study. This research presents examples of successful detections through the suggested models illustrated in Figure 6. It should be noted that the dataset of the 2020 Drone vs. Bird Detection Challenge has a large number of images in which the drones are very small in size and almost indistinguishable from bird images, as can be seen in a few samples provided in Figure 6. All of the investigated models underwent evaluation, and their detection results are showcased alongside the ground truth annotations. Exploring the underlying factors that contribute to the varying performances of each algorithm on distinct sequences is an important area for further study. Upcoming iterations of the contest might assess other dimensions, such as computational complexity (and real-time capabilities), incorporation of supplementary datasets, and the capacity to adapt to entirely distinct backgrounds. These evaluations will help us to better comprehend the various compromises and interactions at play among the multitude of potential tactics and method combinations.

### 3.6. Proposed Model’s Validation on an Unseen Dataset

**Figure 7:**
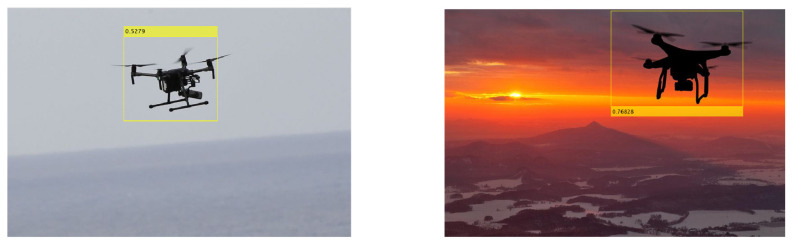

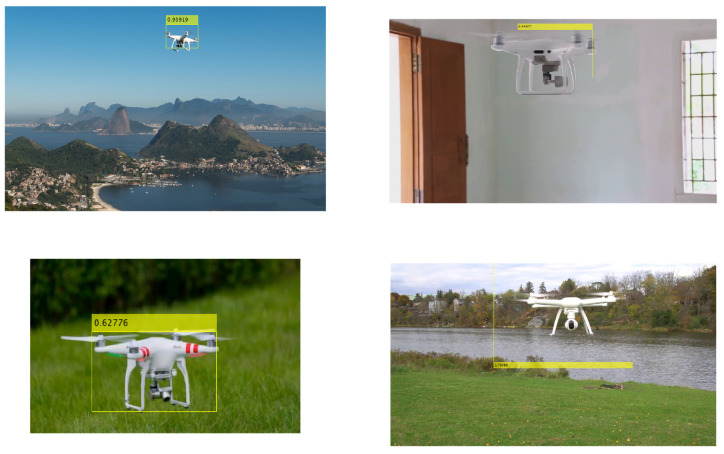
Visual samples displaying qualitative detection of the proposed model on an unseen dataset [36].

To validate the performance of our proposed method, it was investigated on an entirely different dataset. Figure 7 shows the detection results of the proposed model trained using Squeezenet as the backbone on sample test images taken from a different dataset [36]. The detection results prove that the trained model generalized well onto images from an entirely different dataset, as well as under different backgrounds. Only a few instances were not detected in certain images. These results may be reproduced using our developed open-source code provided at the following link: https://github.com/4mbilal/drone_detection (accessed on 3 August 2023).

## 4. Conclusions

The use of drones has become increasingly popular, leading to concerns about their potential use for nefarious purposes, particularly in sensitive locations such as airports. To address this issue, there has been significant interest in developing methods for detecting and recognizing drones. This issue is challenging due to the similarities between the behaviors and appearances of drones and birds in the sky, as well as the high speeds of drones, the presence of crowded backgrounds and hidden areas, lighting issues, and the difficulty of identifying small drones at long distances. To overcome these challenges, this paper proposes a novel deep learning-based approach for detecting and recognizing both drones and birds to prevent unauthorized drone activity. This study utilized a dataset sourced from a real-world dataset made accessible by a consortium of universities and research institutions as a component of the Drone vs. Bird Detection Challenge, held in 2020. Among the various models rigorously evaluated, including SqueezeNet, MobileNetV2, ResNet18, and ResNet50, the SqueezeNet architecture emerged as the standout performer. It exhibited exceptional performance, particularly for medium area ratios, boasting a remarkable average precision (AP) score of 0.770. While the initial results are promising, there remain several avenues for further exploration. In the future, there will be a significant emphasis on harnessing various deep learning networks to effectively differentiate drones from birds. This research, which encompasses diverse drone types, including multi-rotors, helicopters, fixed-wing, and VTOL aircraft, seeks to develop a real-time system capable of detecting, recognizing, and localizing these aerial objects, with integration into onboard systems as the ultimate objective. 

## Figures and Tables

**Table 1:** A summary of the ablation study for selection of the SqueezeNet Hyperparameters.

Hyperparameter		$AP_{0.5:0.9}$	$AP_{0.5}$	$AP_{0.75}$	$AP_{S}$	$AP_{M}$	$AP_{L}$
MiniBatch Size	1	0.23581	0.12513	0.12513	0.50952	0.28393	0.013993
	**2**	**0.327**	**0.219**	**0.219**	**0.631**	**0.770**	**0.216**
	4	0.26337	0.14832	0.14832	0.55419	0.639	0.083013
	8	0.22958	0.098146	0.098146	0.52013	0.48746	0.03198
	16	0.14726	0.037511	0.037511	0.38704	0.21077	0.001357
L2 Regularization	0.00025	0.088229	0.014326	0.014326	0.25557	0.001569	0
	**0.0005**	**0.327**	**0.219**	**0.219**	**0.631**	**0.770**	**0.216**
	0.001	0.25423	0.13265	0.13265	0.54114	0.69698	0.087404
Momentum	0.95	0.162	0.095	0.095	0.51	0.67	0.182
	**0.9**	**0.327**	**0.219**	**0.219**	**0.631**	**0.770**	**0.216**
	0.85	0.17874	0.05453	0.05453	0.44449	0.31732	0.010222

**Table 2:** A comparison between the two architectures across different overlap thresholds, area ratios, and operation times.

Backbone	*T_i_*	*T_t_*	*T_s_*	$AP_{0.5:0.9}$	$AP_{0.5}$	$AP_{0.75}$	$AP_{S}$	$AP_{M}$	$AP_{L}$
SqueezeNet	0.319	332.935	**0.061**	**0.327**	0.219	0.219	0.631	**0.770**	0.216
ResNet18	0.455	117.209	**0.105**	**0.237**	0.082	0.082	0.557	**0.739**	0.264

where *T_i_* is average time per single iteration (sec), *T_t_* is time for training the network (min), *T_s_* is time for single detection (sec), $AP_{0.5:0.9}$ represents the average precision calculated using an overlap threshold that ranges from 0.5 to 0.9, and $AP_{S},\;AP_{L},\;\mathrm{and}\;AP_{M}$ denote the average precision values for small, large, and medium area ratios, respectively.

**Table 3:** The performance of the two architectures across different overlap thresholds, area ratios, and operation time.

Backbone	*T_i_*	*T_t_*	*T_s_*	$AP_{0.5:0.9}$	$AP_{0.5}$	$AP_{0.75}$	$AP_{S}$	$AP_{M}$	$AP_{L}$
ResNet50	1.078	273.729	**0.129**	**0.254**	0.104	0.104	0.579	**0.610**	0.214
MobileNetV2	0.861	219.666	**0.111**	**0.166**	0.036	0.036	0.442	**0.485**	0.079

**Table 4:** Differences in results between this study and prior research.

Method	SqueezeNet	MobileNetV2	ResNet18	ResNet50	(Coluccia et al., 2021) [35]
***AP* (%)**	0.770	0.485	0.739	0.610	52.2

## Data Availability

Publicly available datasets were analyzed in this study. This data can be found here: https://wosdetc2021.wordpress.com/drone-vs-bird-detection-challenge/.
